# Structural Characterization and Bioactivity of a Titanium(IV)-Oxo Complex Stabilized by Mandelate Ligands

**DOI:** 10.3390/molecules29081736

**Published:** 2024-04-11

**Authors:** Barbara Kubiak, Tadeusz Muzioł, Grzegorz Wrzeszcz, Aleksandra Radtke, Patrycja Golińska, Tomasz Jędrzejewski, Sylwia Wrotek, Piotr Piszczek

**Affiliations:** 1 Department of Inorganic and Coordination Chemistry, Faculty of Chemistry, Nicolaus Copernicus University in Toruń, Gagarina 7, 87-100 Toruń, Poland; tadeuszmuziol@wp.pl (T.M.); wrzeszcz@umk.pl (G.W.); aleksandra.radtke@umk.pl (A.R.); 2 Department of Microbiology, Faculty of Biological and Veterinary Sciences, Nicolaus Copernicus University in Toruń, Lwowska 1, 87-100 Toruń, Poland; golinska@umk.pl; 3 Department of Immunology, Faculty of Biological and Veterinary Sciences, Nicolaus Copernicus University in Toruń, Lwowska 1, 87-100 Toruń, Poland; tomaszj@umk.pl (T.J.); wrotek@umk.pl (S.W.)

**Keywords:** titanium(IV)-oxo complex, α-hydroxyacid, mandelic acid, crystal structure, antimicrobial activity, cytotoxicity

## Abstract

Research on titanium-oxo complexes (TOCs) is usually focused on their structure and photocatalytic properties. Findings from these investigations further sparked our interest in exploring their potential biological activities. In this study, we focused on the synthesis and structure of a compound with the general formula [Ti_8_O_2_(O^i^Pr)_20_(man)_4_] (**1**), which was isolated from the reaction mixture of titanium(IV) isopropoxide with mandelic acid (Hman) in a molar ratio of 4:1. The structure (**1**) was determined using single-crystal X-ray diffraction, while spectroscopic studies provided insights into its physicochemical properties. To assess the potential practical applications of (**1**), its microcrystals were incorporated into a polymethyl methacrylate (PMMA) matrix, yielding composite materials of the type PMMA + (**1**) (2 wt.%, 5 wt.%, 10 wt.%, and 20 wt.%). The next stage of our research involved the evaluation of the antimicrobial activity of the obtained materials. The investigations performed demonstrated the antimicrobial activity of pure (**1**) and its composites (PMMA + (**1**)) against both Gram-positive and Gram-negative strains. Furthermore, MTT tests conducted on the L929 murine fibroblast cell line confirmed the lack of cytotoxicity of these composites. Our study identified (**1**) as a promising antimicrobial agent, which is also may be use for producing composite coatings.

## 1. Introduction

The wide interest in titanium(IV)-oxo complexes (TOCs) is associated with their structural diversity and photocatalytic activity [1,2]. These compounds have found applications in diverse technologies, including hydrogen production or water purification [3,4]. Previous research led to the synthesis of TOCs with cores containing from 2 to 44 titanium atoms [5,6,7,8,9,10,11,12], with the largest group consisting of compounds with four- and six-nuclear cores [13,14,15,16,17]. The cores {Ti_a_O_b_} of TOCs can be stabilized by alkoxide groups, as well as carboxylate or phosphonate ligands, although β-diketonate, β-ketoester, and sulfonate ligands have also been employed [5,18,19]. The varied possibilities of carboxylate ligand coordination with titanium atoms [20] explain the fact that most of the works focus on investigations of Ti(IV)-oxo complexes stabilized with alkoxide and carboxylate ligands. Notably, the type of carboxylate ligand has a significant influence both on the {Ti_a_O_b_} core structure and the oxo complex photocatalytic activity [5,7,21,22,23,24,25,26].

Interest in Ti(IV)-oxo complexes also stems from their potential biomedical applications. Studies on the utilization of TOCs in photodynamic therapy [27] and as antimicrobial agents [5,13,24,28,29,30] are particularly significant. With the ongoing search for materials possessing bactericidal properties and the ability to prevent microbial growth, our research has concentrated on the latter aspect. The analysis of literature reports suggests that research on this issue is developing in three main directions. The first involves the introduction of a heteroatom into the core structure, resulting in the synthesis of compounds containing {AgTi-oxo} or {AgTi} cores, which demonstrate favorable optical, antibacterial, and photothermal properties [29,30]. Luo et al., in their study on a complex featuring a {Ag_9_Ti_4_} core stabilized by salicylate ligands, proved that its bacteriostatic efficacy against strains of *Staphylococcus aureus* and *Escherichia coli* was 94.51% and 95.42%, respectively, surpassing that of a comparable complex with a {Ag_2_Ti} core [30]. The improvement in the biocidal properties of the complex with the {Ag_9_Ti_4_} core was achieved by the formation of a hydrogel {Ag_9_Ti_4_-gel}.

Another approach to leveraging the antibacterial properties of Ti(IV)-oxo complexes was introduced in the research of Svensson et al. [13]. In their study of the tetranuclear Ti(IV)-oxo complex ({Ti_4_O_2_} core) stabilized with triclosan ligands, they capitalized on the compound’s high susceptibility to hydrolysis processes, facilitating the release of an antibacterial agent (triclosan). According to this theory, Ti(IV)-oxo complexes are considered as carriers of bactericidal agents. The third strategy involves harnessing the photocatalytic activity of TOCs and their capability to generate reactive oxygen species (ROS) [25,31]. In this case, the mechanism of biocidal action is associated with oxidative stress induced by the generated ROS. Our previous investigations confirmed the biocidal activity of {Ti_4_O_2_} clusters stabilized with carboxylate ligands (such as 4-aminobenzoic acid, 4-hydroxybenzoic acid, and 9-fluorene carboxylate) [5,24,25]. The analysis of the electron paramagnetic resonance (EPR) spectra of these compounds confirmed the generation of O_2_^−^ and O^−^ oxygen anions on the surface of both pure oxo complexes and composites prepared by dispersing the complexes in a poly(methyl methacrylate) (PMMA) matrix. It is worth noting that the {Ti_a_O_b_} core structure also influenced the antimicrobial activity of composites containing Ti(IV)-oxo complexes. Studying oxo complexes with different core structures stabilized by 9-fluorene carboxylate ligands allowed for the observation of this effect. It was found that the biocidal activity of samples with {Ti_6_O_4_} and {Ti_4_O_2_} cores was higher than that of {Ti_6_O_6_} and {Ti_3_O} systems, when irradiated with visible light [5]. 

To enhance the biocidal properties of synthesized TOCs, we decided to incorporate α-hydroxy carboxylate ligands, known for their antimicrobial and anti-inflammatory properties [32,33,34], into their structure. Our goal was to develop a new, durable material with antimicrobial traits which were responsive to light exposure in the UVA and visible range. The introduction of carboxylate ligands possessing α-hydroxy groups to the structure of TOCs increases their possibilities of coordinating with Ti(IV), resulting in the formation of more structurally stable systems in comparison to those of compounds stabilized by carboxylate groups [5,26,35]. Previous investigations have proven that the photocatalytic properties of TOCs may be stabilized with the addition of α-hydroxy carboxylate ligands such as salicylate, 4-chlorosalicylate, 1-hydroxynaphthoate, citrate, and 2,5-dihydroxybenzoate [14,26,35,36,37,38]. Therefore, incorporating such ligands into the structure of oxo complexes is also expected to yield systems with biocidal activity. 

Considering the widespread application of mandelic acid (Hman) as a bactericidal agent in the cosmetic industry, its introduction to the structure of TOCs was intriguing [39,40]. Previous research regarding mandelic acid properties showed that prolonged skin exposure to Hman absorption may lead to redness, dryness, and excessive skin exfoliation [41,42]. Hence, investigations carried out in recent years has focused on the synthesis and evaluation of the biocidal action of Hman salts and their complex compounds [43,44]. The compounds synthesized in the reaction of titanium isopropoxide with mixtures of mandelic and benzohydroxamic acid [45] or mandelic and phenylphosphonic acid [46] typically form dimeric structures, wherein two titanium atoms ({Ti_2_}) are connected solely by mandelic ligands (man). Schetter et al. synthesized titanium complexes with multinuclear cores, particularly {Ti_2_}, {Ti_6_}, {Ti_9_}, stabilized by mandelic and tert-butoxide ligands [47]. Mandelic ligands play a crucial role in the formation of {Ti_2_} dimer units by binding to metal atoms via carboxyl and hydroxyl groups. This mode of coordination also facilitates the construction of larger {Ti_6_} and {Ti_9_} systems. It is worth noting that the described compounds have no {Ti_a_O_b_} cores, so the oxo complex we have synthesized is completely new, from a structural point of view.

In this paper, we present the results concerning the synthesis of a structurally stable Ti(IV)-oxo complex with the general formula [Ti_8_O_2_(O^i^Pr)_20_(man)_4_] (**1**). The novel {Ti_8_O_2_} core exhibits a unique topology characterized by a plethora of labile isopropoxide ligands and a limited number of oxo bridges. Our objective was to evaluate the biocidal activity of the synthesized compound (**1**) and the composite films formed by dispersing (**1**) in the PMMA matrix (PMMA + (**1**)).

## 2. Results

### 2.1. Structure of (***1***) Oxo Complex

**Figure 1:**
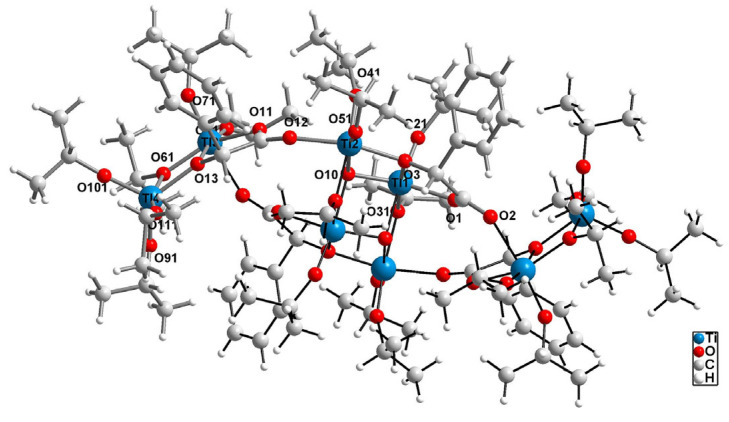
Structure of [Ti_8_(μ_3_-O)_2_(μ-O^i^Pr)_4_(O^i^Pr)_16_(man)_4_] (**1**) as a ball and stick model, with hydrogen atoms omitted for the clarity of the figure. The structure presents only the main conformations. Atom labels for the titanium and oxygen atoms are provided. The asymmetric area is presented with bonds shown in medium grey, and the area related to the inversion center is shown in black.

The octanuclear cluster {Ti_8_O_2_} of the oxo complex (**1**) is formed around a central tetranuclear {Ti_4_O_2_} unit, in an unprecedented manner, with two lateral dimers {Ti_2_} attached to it via O1 and O11 mandelate ligand bridges (Figure 1). In these lateral dimers, the Ti3 and Ti4 cations are connected by an O61 isopropionate anion and an O13 hydroxyl group from the O11 mandelate anion, resulting in a very short intermetalic distance between them, measuring 3.2486(15) Å (Table S1). The central {Ti_4_O_2_} core consists of two Ti1 and two Ti2 cations, forming a slightly distorted rhombus, with Ti1-Ti2 distances of 3.1036(10) and 3.1940(10) Å, respectively. This arrangement is stabilized by two O10 μ_3_-oxo bridges connecting two Ti1 cations and one Ti2 cation, along with two O31 isopropionate anions and two O3 hydroxyl groups from the O1 mandelate anion. The O10 oxo group forms one short bond and two much longer bonds, leading to a topology distinct from those of the previously reported {Ti_4_O_2_} cores, in which titanium cations are coupled by one µ_4_-oxo and one µ-oxo bridge [7] (Figure S1). Ti(IV) titanium cations exhibit significant differences in coordination number and coordination sphere content (refer to Table S2). Ti1, Ti2, and Ti3 are found in an octahedral environment, whereas the outermost Ti4 cation is found in pentacoordinated, significantly distorted, trigonal bipyramid coordination sphere (τ5 = 0.60) [48]. Mandelate ligands are invovled in bridging titanium cations. Moreover, they also form chelate rings, with Ti1 and Ti3 cations. In those rings, an enhanced charge concentration is observed (Figure S2).

**Figure 2:**
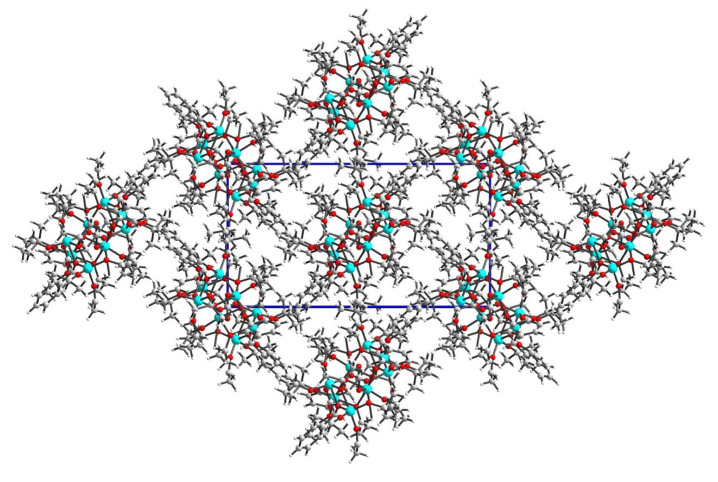
Crystal network of (**1**) along the c axis shows ab layers.

**Figure 3:**
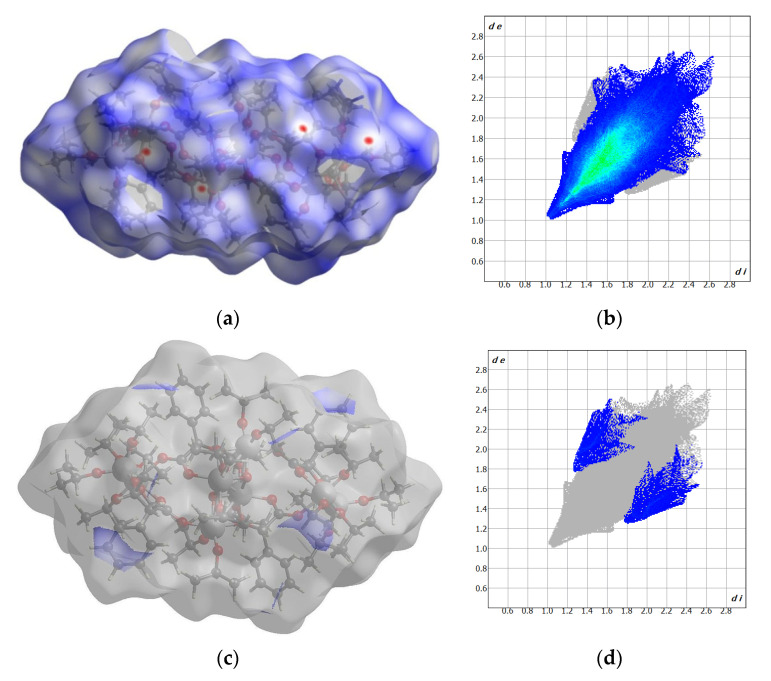
Hirshfeld surfaces and fingerprints of selected interactions created in the crystal network of (**1**): Hirshfeld surface (**a**) and fingerprint (**b**) for H…H (96.8%); Hirshfeld surface (**c**) and fingerprint (**d**) for H…C (3.2%). In brackets, the surface area is included as a percentage of the total surface area.

The crystal network does not exhibit any cavities containing uniformly distributed octanuclear clusters (Figure 2). Hirshfeld surface analysis reveals that weak interactions dominate the landscape of the formed interactions (Figure 3). Specifically, H…H interactions constitute 96.8% of the intermolecular contacts, with the remaining 3.2% attributed to H…C contacts. Despite careful analysis, no intermolecular hydrogen bonds were observed, while several intramolecular C-H…O hydrogen bonds were identified.

These results suggest that the oxygen atoms are buried and inaccessible to the hydrogen atoms from the adjacent clusters. Moreover, the high propanalate/mandelate ratio (5:1) results in only one π–π interaction, occurring between the strongly inclined C14 phenol ring and the Ti3O11 five-membered chelate ring. In summary, we synthesized a stable complex with the unprecedented core structure {Ti_8_O_2_}, which may interact with biomolecules in the cell via external and easily accessed unsaturated Ti4 cation, as well as through weak non-covalent van der Waals forces.

### 2.2. Spectral Cheracterization of (***1***) and Its Composite with Poly(methyl methacrylate)

**Figure 4:**
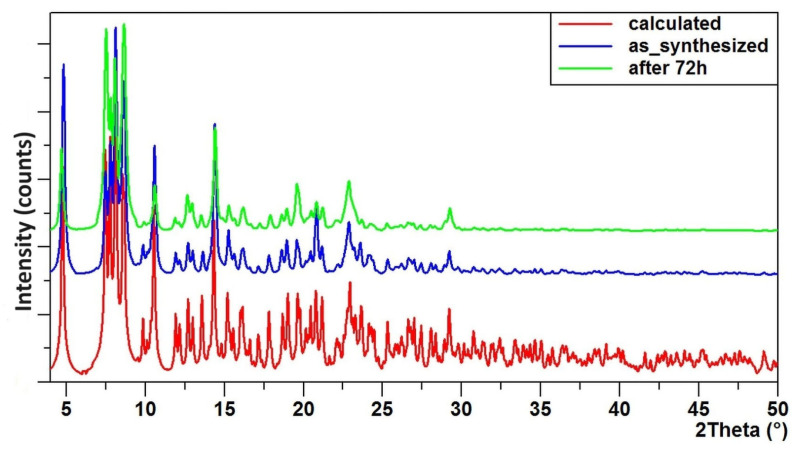
The powder diffractograms, calculated using our model (red), the synthesized complex (blue), and the sample after being immersed in distilled water for 72 h (green), in the range of 4–50° 2θ.

**Figure 5:**
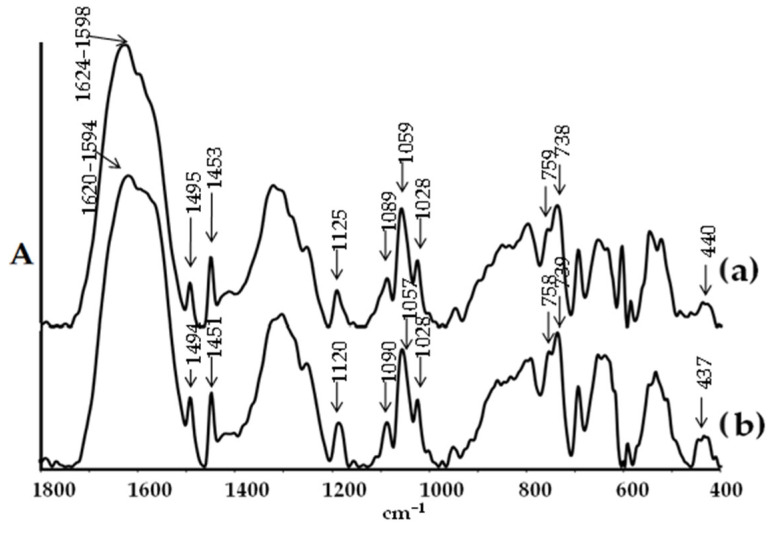
IR spectrum of structure (**1**) before (**a**) and after (**b**) 72 h contact with water.

The objective of the spectral studies was to determine the structural stability, including susceptibility to hydrolysis processes, and the physicochemical properties of both the complex and its composite formed by dispersing micrograins of this compound in a non-toxic poly(methyl methacrylate) matrix (PMMA + (**1**)). Research on composite systems has aimed to assess their practical potential as antimicrobial coatings. To this end, PMMA + (**1**) composite samples were prepared, with 2 wt.%, 5 wt.%, 10 wt.%, and 20 wt.%. Figure 4 illustrates the XRD patterns of the (**1**) sample, recorded before and after immersion in distilled water for 72 h. Both diffractograms were compared to the pattern calculated in Ref. [49]. Analysis of the XRD data revealed no significant differences in the patterns, before and after immersion in water. The resemblance between these diffractograms indicates that the core structure was preserved, and no significant transformation towards crystalline TiO_2_, amorphous forms, or other crystalline species related to rearrangement of the titanium co-ordination spheres and topology, was detected. The structural stability of (**1**) was further confirmed using IR spectroscopy (Figure 5). The comparison of the IR spectra of samples, before and after immersion in water, showed no changes. There were no alterations in the intensity and position of the bands within the ranges of (a) 1624–1450 cm^−1^, which are associated with the vibrations ν(CC), ν_as_(COO), and ν_s_(COO), (b) 900–1200 cm^−1^, assigned to the ν(O-CR) vibrations, and (c) bands appearing below 900 cm^−1^, indicated titanium-oxo bridges.

**Figure 6:**
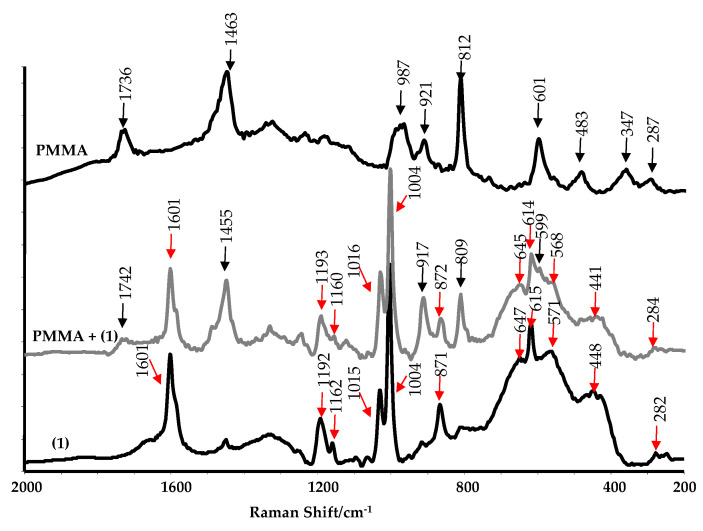
Raman spectra of (**1**), PMMA + (**1**) 20 wt.%, and pure PMMA.

The presence of (**1**) in the polymer matrix was confirmed using SEM EDX (Table 1). Titanium peaks are observed in each composite sample, and the percentage of titanium increases with the (**1**) sample concentration. To verify the absence of structural changes in (**1**) upon incorporation into the PMMA matrix, we compared the Raman spectra of the oxo complex (**1**), the composite of PMMA + (**1**) 20 wt.%, and pure PMMA polymer (Figure 6). The results obtained suggest that the structure of the compound remains unchanged upon its introduction into the matrix.

The EPR spectra of (**1**) powders and cut composite foil were recorded to identify paramagnetic species on the surface of the synthesized materials (Table 2 and Figure S2). The pure PMMA polymer exhibited no EPR signal. Paramagnetic centers were detected in all the samples of the PMMA + TOCs composites, as well as in the spectrum of pure (**1**). Generally, the signals in the EPR spectra were weak, particularly for composites with a low (2,5,10 wt.%) admixture of titanium(IV)-oxo complexes (although ROS signals were detected, see Table 2). Consequently, only the EPR spectra of the pure (**1**) sample and the composite sample containing 20 wt.% of titanium(IV)-oxo complex grains were analyzed in detail. The spectra registered for pure (**1**) and the composite of PMMA + (**1**) 20 wt.% are presented in Figure S3. The analysis of the EPR spectra of the aforementioned samples demonstrated that natural photoexcitation of their surfaces, i.e., generated without additional UV–Vis lamp radiation, led to the formation of ROS. Additionally, it is worth noting that both O^−^ and O_2_^−^ paramagnetic species were generated by the PMMA + TOCs composite. Interestingly, the O_2_^−^ radical was also generated in the pure substrate.

### 2.3. Antimicrobial Activity of (***1***) and Its Composites

The subsequent phase of our research involved evaluating the biocidal activity of both (**1**) and the PMMA + (**1**) composite. These assessments were conducted against Staphylococcus aureus and Escherichia coli bacteria, as well as *Candida albicans fungi*. The results, as depicted in Table 3, reveal that the complex (**1**) exhibited significant antimicrobial activity, even at low concentrations. According to the ISO 22196:2011 standard [50], the suspensions exhibit a biocidal effect when the R value is ≥3. In the case of (**1**) suspensions, the R value for Gram-negative bacteria ranged from 5.7 to 6.0, while for Gram-positive bacteria, it varied from 4.2 to 6.2. Microbiological activity against *Candida albicans* was only apparent with a 20% (*w*/*v*) addition of (**1**). Non-porous surfaces are considered bactericidal when R ≥ 2. Microbiological tests of composite samples (PMMA + (**1**)) exhibited outstanding activity against both *E. coli* and *S. aureus* bacteria across all tested concentrations of TOCs (2 wt.%, 5 wt.%, 10 wt.%, and 20 wt.%), as detailed in Table 3. Even at a modest (**1**) content of 2 wt.%, the composite displays effective activity against E*. coli* bacteria (R ≥ 2). In terms of *S. aureus* bacteria, the R values ranged from 4.7 to 5.1. It is noteworthy that the tested composites do not diminish the count of *C. albicans* cells.

### 2.4. Cytotoxicity of PMMA + (***1***) Composites

**Figure 7:**
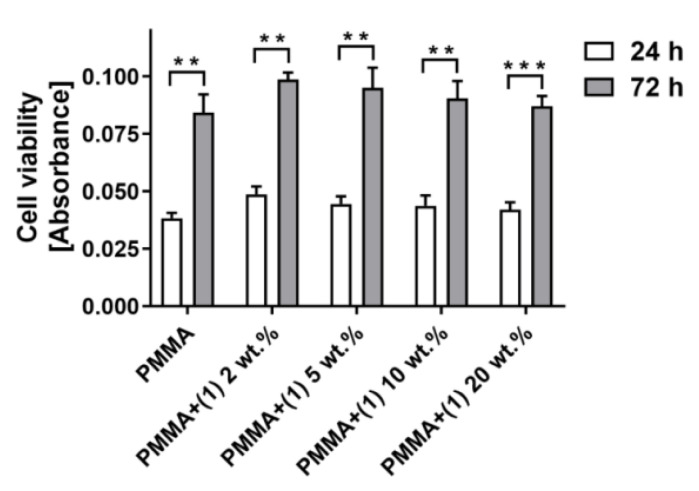
The viability of L929 fibroblasts growing on the surfaces of the PMMA reference samples and the PMMA specimens containing mandelic ligands at a concentration of 2 wt.%, 5 wt.%, 10 wt.%, and 20 wt.%. The cells were cultured on the samples for 24 and 72 h. The absorbance values are expressed as means ± S.E.M of four independent experiments. Asterisks indicate significant statistical differences in the cell viability between 24 and 72 h (*** *p* < 0.001; ** *p* < 0.01).

The potential cytotoxicity of the tested specimens was assessed using MTT assays and analysis of the SEM images. The purpose of this study is to determine whether compound (**1**) can damage mammalian cells. A material is considered non-cytotoxic if cell viability is greater than 70%. The results of the MTT assays showed that the level of cell viability measured for the reference PMMA samples was comparable to the values received for specimens containing mandelic ligands in all tested concentrations. This effect was observed for both 24 and 72 h of incubation time. Importantly, these results also demonstrated that with an increase in the incubation time, more L929 fibroblasts proliferated on all the tested specimens (Figure 7).

**Figure 8:**
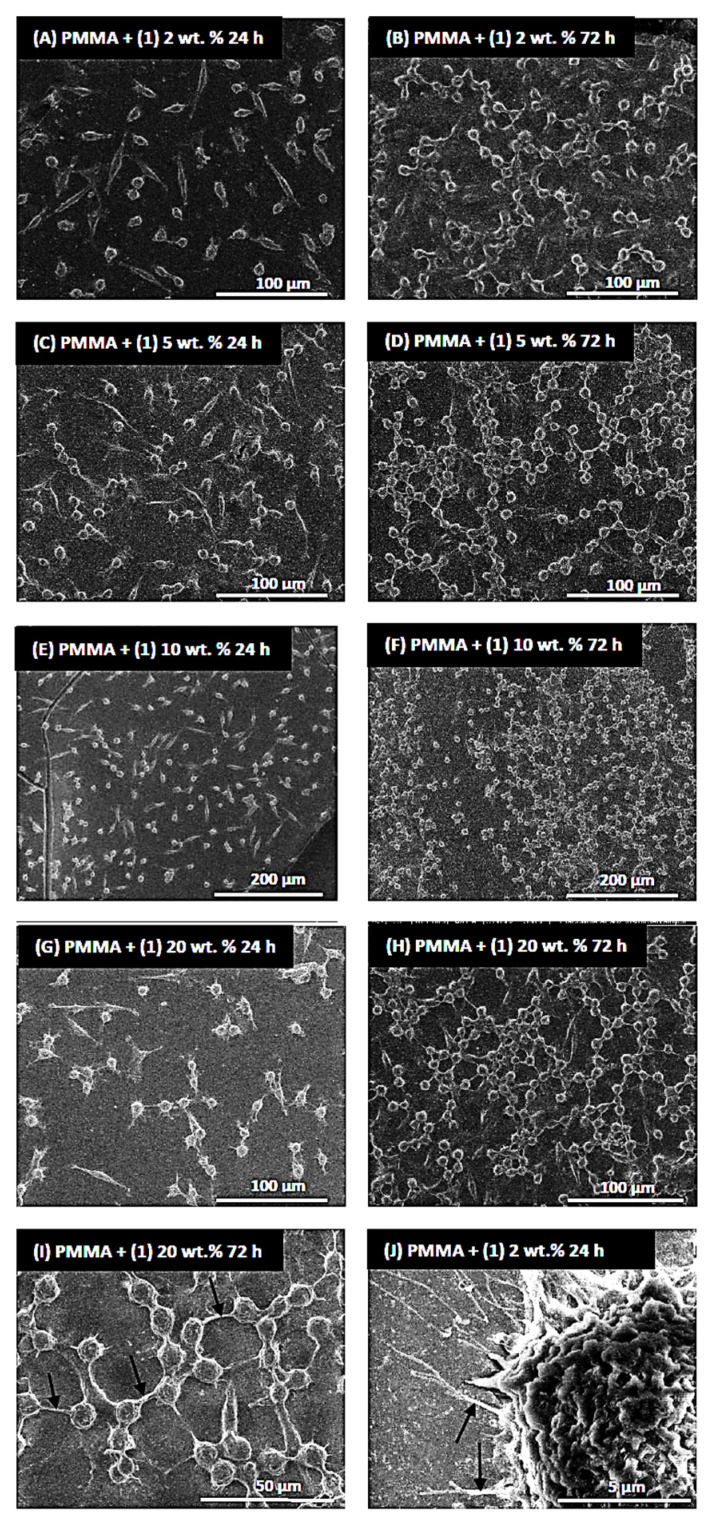
Scanning electron microscopy images of L929 cells cultured on the surfaces of PMMA specimens containing mandelic ligands at a concentration 2 (**A**,**B**,**J**), 5 (**C**,**D**), 10 (**E**,**F**), and 20 wt.% (**G**,**H**,**I**). The type of samples, incubation time, and scales of the images are presented in the figures. Black arrows in (**I**) indicate cytoplasmic projections spread between the cells, whereas those in (**J**) present cytoplasmic projections attaching the cells to the surface.

Figure 8 presents selected images of L929 cells cultured on the PMMA specimens containing mandelic ligands at a concentration of 2 (A, B, J), 5 (C, D), 10 (E, F), and 20 wt.% (G, H, I). Firstly, the analysis of these micrographs confirmed the results from the MTT assays, indicating that L929 fibroblasts effectively attached to the surfaces, and the number of the growing cells increased over time. After 72 h, the fibroblasts covered the sample surface area at a significantly higher density, which is especially visible in different magnifications of electron microscopy photos (compared Figure 8E,F).

Importantly, the elongated shape of the cells was already observed after 24 h of culturing, indicating their normal adhesion, with few cytoplasmic projections at the cell edges, which also allowed cells to attach to the surface of the specimens (Figure 8J). After 72 h, the number of cytoplasmic projections at the cell periphery had increased, and after entering into closer proximity to the neighboring cells, they started forming cell–cell contacts (Figure 8I). The cytoplasmic projections, called filopodia, play a fundamental role in cell attachment, migration, proliferation, and cell–cell interaction [51,52]. The results from the MTT assays and the SEM analysis showed that the tested specimens containing MA did not affect the morphology of the L929 fibroblasts and did not reduce their viability.

## 3. Discussion

### 3.1. Structure and Physicochemical Properties of (***1***) and Its Composite PMMA + (***1***)

The crystals with the general formula [Ti_8_O_2_(O^i^Pr)_20_(man)_4_] (**1**) were obtained at room temperature from a reaction mixture containing titanium isopropoxide and mandelic acid in a 4:1 molar ratio, using tetrahydrofuran as a solvent. Single crystal X-ray diffraction analysis confirmed that the structure of (**1**) consists of a {Ti_8_O_2_} core stabilized by isopropoxide and mandelate ligands (Figure 1). Previous studies have reported the occurrence of {Ti_8_O_8_} cores in octanuclear Ti(IV)-oxo complexes, composed of eight-membered rings, where Ti atoms are surrounded by μ-O and μ-carboxyl bridges [53,54,55,56,57]. Czakler et al. synthesized a compound with a {Ti_8_O_2_} core consisting of two {Ti_4_O} units bridged by two μ_3_-O atoms [58]. 

The structure of (**1**) is more intricate, as its core comprises a central {Ti_4_O_2_} unit and two lateral dimers ({Ti_2_}). This reported structure presents a novel, unprecedented core among titanium-oxo complexes (TOCs), stabilized by two μ_3_-O bridges and mandelate anions forming bridges, and for Ti1 and Ti3, a five-membered chelate ring. To verify the structural stability of (**1**), both in aqueous solutions and after its integration into the polymer matrix, spectroscopic analyses were conducted on powder samples after contact with water (employing XRD and IR methods) and samples of the PMMA + 20 composite (**1**) (with Raman spectra recorded). 

TOCs are recognized for their vulnerability to structural destabilization and hydrolysis. Compounds prone to hydrolysis find limited utility, particularly in applications involving exposure to aqueous environments. The existing literature suggests that incorporating carboxylates, phenols, phosphonates, and catechols into the coordination sphere can bolster the stability of such structures [12,59,60,61]. Our research affirms that the obtained compound maintained its structure when exposed to an aqueous environment. This result is supported by the absence of notable alterations in the X-ray diffraction patterns and IR spectra, before and after exposure to water. Examination of the Raman spectra of PMMA + (**1**) 20 wt.% composite samples revealed that the compound (**1**) maintained its structure subsequent to its integration into the polymer matrix.

The pivotal focus of this stage of the research centered on evaluating the photoactivity of both the pure oxo complex and its composites. Utilizing electron paramagnetic resonance (EPR) facilitated the detection of reactive oxygen species (ROS) generated on the surface of samples previously exposed to visible light. Considering the literature data, it is reasonable to assume that upon photoexcitation of the samples, Ti(III) and ROS would be initially generated on their surfaces [62,63,64,65,66]. 

The formation of O_2_^−^ and O^−^ type paramagnetic centers involves electron removal and stabilization of the radical formed by reducing Ti(IV) to Ti(III) [62,63]. The maximum g-factor for O_2_^−^ exceeds that of O^−^ species [64]. In the EPR spectrum of (**1**), characteristic anisotropic signals for the O_2_^−^ radical were identified (Table 2, Figure S2a). Detecting titanium(III) is not straightforward; in this case, a weak and broad signal, nearly at the noise level, may be present (Figure S2a, Table 2). The EPR spectra of PMMA + (**1**) composites are more intricate and contain numerous overlapping lines (Figure S2). The spectra of all the composites are akin, except for variations in intensity. Weaker lines are indiscernible for samples containing minor admixtures (2 and 5%) of the titanium(IV) complex. In the spectrum of PMMA + (**1**) 20% wt.%, distinct signals of both ROS, i.e., O_2_^−^ and O^−^, as well as Ti(III), are evident (Table 2, Figure S2b). Some lines, particularly for g_2_ and g_3_ of both oxygen species, may overlap. Individual lines are relatively broad due to the high content of (**1**) and potentially high paramagnetic center concentrations, resulting in line broadening via exchange interactions. In contrast to similar TOCs composites with PCL [25,67], Ti(III) was discerned on the surface of the investigated composite material (Table 2, Figure S2b). Due to a positive spin–orbit coupling constant, Ti(III) exhibits EPR signals at g-values below 2.0 [25,62,63,64,65,66,67]. The first signal, with a g-factor of 1.992, is clearly visible, while the second, with a lower g-factor of approximately 1.972, is very weak and broad.

### 3.2. Antimicrobial Activity of (***1***) and Its Composite PMMA + (***1***)

Elucidating the biocidal attributes of the synthesized oxo complex (**1**) and its composite PMMA + (**1**) involves considering two factors: (i) the photocatalytic capability of the oxo complex, and (ii) the structural factors, particularly the formation of chelate rings. The photocatalytic prowess of (**1**) is associated with the generation of reactive oxygen species (ROS) upon exposure to visible light, as confirmed by EPR tests. Introducing ROS to a bacterial cell induces oxidative stress, leading to the impairment of cellular lipids, proteins, and nucleic acids, ultimately resulting in the demise of the microorganism [68,69,70,71].

The antimicrobial efficacy can also be linked to the compound’s structure, especially the formation of chelate rings and surface hydrophobicity (as observed in the Hirshfeld surface). According to Tweedy’s chelation theory and Overtone’s concept, coordination diminishes the metal ion’s polarity by overlapping the ligand orbitals and partially sharing the positive charge with the donor groups [72]. The enhanced delocalization of π-electrons in the chelate ring augments the compound’s lipophilic nature. This increased lipophilicity promotes the solubility of lipids in the cell membrane upon contact with the oxo complex grains, leading to irreversible damage to the membrane and cell functions [72].

Literature reports suggest a correlation between electron density and antimicrobial activity, with chelation increasing the electron density of the central ion [73]. It should be emphasized that the synergy of mechanisms involving the generation of ROS and effects related to the presence of a chelating ring can only be considered as the primary mechanism in the case of the antimicrobial action of (**1**), where the entire surface of the oxo complex grain comes into contact with the microbial suspension. However, in composite materials, the grains are surrounded by a polymeric matrix exhibiting weaker biocidal activity, solely due to ROS generation on their surface. This is confirmed by the data analysis presented in Table 3, indicating that the activity of the PMMA + (**1**) composite is weaker compared to that of the pure compound.

Studies on the antimicrobial activity of mandelic acid (Hman) revealed that the minimum inhibitory concentration (MIC) required to inhibit the growth of *S. aureus* and *E. coli* strains fell within the range of 0.18–0.23% [43,74]. In the case of crystalline powder tests of (**1**), the mandelic ligand content in the suspension of the studied sample was 0.013%, 0.034%, 0.069%, and 0.14% for concentrations of 2%, 5%, 10%, and 20% (*w*/*v*), respectively. This suggests that the antibacterial activity of (**1**) against *S. aureus* and *E. coli* strains is superior to that of mandelic acid.

When evaluating the biocidal properties of the tested samples, it is essential to consider that, according to ISO standards, a suspension is deemed bactericidal when 99.90% or more of the microorganisms are inhibited (reduction index (R) ≥ 3), while for non-porous surfaces, the reduction index is ≥2. It seems that the incorporation of (**1**) grains into a polymer matrix resulted in a decrease in antimicrobial activity when compared with that of suspensions, but biocidal activity was still achieved at the required level, according to ISO 22196:2011 standard (Table 3). 

An interesting comparison can be made with the results obtained by Luo et al. for compounds containing {Ag_9_Ti_4_} cores, as well as type {Ag_9_Ti_4_}-gel hydrogel samples. Antimicrobial activity assessments showed that {Ag_9_Ti_4_} crystals exhibited antibacterial activity on the level of 94.51% for *S. aureus* and 95.42% for *E. coli*, respectively [30]. For comparison, our oxo complex (**1**) showed an inhibitory effect of >99.99% for both strains of the tested samples containing 2% (*w*/*v*) in the studied suspension. The hydrogel of {Ag_9_Ti_4_}-gel exhibited bactericidal effects of 99.6% against *S. aureus* and 99.95% against *E. coli* [30]. The authors suggest that the increase in antimicrobial activity is a result of the synergistic effect of the {Ag_9_Ti_4_} cluster and polydopamine, added as a cross-linking agent in hydrogel formation. In the case of our composites, even a 2% addition of TOCs reduced the presence of *S. aureus* by 99.00% and *E. coli* by 99.99%. This is in line with the ISO standard for bactericidal properties, which states that the number of microorganisms must be reduced by ≥99% (R ≥ 2).

### 3.3. Cytotixity of PMMA + (***1***) Composites

Testing the cytotoxicity of the obtained materials was a very important aspect of our study, from the application point of view. In addition to antimicrobial properties, the obtained surfaces should not exhibit cytotoxicity towards fibroblast cells. This guarantees safety in case of contact with skin. We presume that the topology affects the cytotoxic activity. The exact mechanism of titanium cluster cytotoxicity is not known, and it is not clear if it is related to DNA intercalation [75]. Studies show that the cytotoxic effect is related to the stability of the compound. Hydrolyzed molecules are characterized by strong cytotoxic activity. L(OEt)Ti-O-Ti(OEt)L, as a product of the partial hydrolysis of [L(OEt)_2_Ti], shows strong cytotoxicity, despite bulky substituents [76]. The compound we obtained is characterized by high stability and low cytotoxicity, which confirms this theory.

## 4. Materials and Methods

### 4.1. Materials

Titanium(IV) isopropoxide was acquired from Sigma-Aldrich, Inc. (St. Louis, MO, USA), while mandelic acid was purchased from Warchem LLC (Warsaw, Poland). Both compounds were used without further purification. Before application, tetrahydrofuran (THF) underwent distillation and was then stored in an argon atmosphere. The synthesis of Ti(IV)-oxo complexes was conducted under an inert gas atmosphere (Ar) at room temperature (RT).

### 4.2. Synthesis of Ti(IV) Oxo-Complex (α-TOCs) and Preparation of PMMA/TOC Composites

#### 4.2.1. The Synthesis of [Ti_8_O_2_(O^i^Pr)_20_(man)_4_] (**1**)

A total of 0.13 g of mandelic acid (0.875 mmol), 1 mL of titanium(IV) isopropoxide (3.5 mmol), and 2 mL of THF were mixed, yielding a clear yellow solution. Crystals of (**1**) appeared after 3 days. The yield based on acid was 66% (0.62 g). Anal. Calc. for C_92_H_168_O_34_Ti_8_:C, 50.18; H, 7.63; Ti,17.45. Found: C, 50.09; H, 7.58; Ti, 17.54. ^13^C NMR (solid state, 295 K, δ[ppm]): 9.75 (CH3), 25.4, 30.1 (CH), 60.1, 71.3 ((Ph)C(Ph)), 90.7, 130.7, 139.6, 182.3, (C(Ph)), 198.3, 209.4 (COO).

#### 4.2.2. PMMA/TOCs Composites Preparation

PMMA/TOCs composites were prepared by adding an appropriate amount of the TOC (ca. 0.025, 0.062, 0.12, or 0.25 g of (**1**) dispersed in 5 mL of THF) to the poly(methyl methacrylate) (PMMA) solution (1.0 g of PMMA per 10 mL of THF). After 150 min in an ultrasonic bath, the dispersions were poured into a glass Petri dish and held at RT in a glove box to evaporate the THF. As a result, materials containing 2, 5, 10, and 20% of TOC were obtained.

### 4.3. Analytical Methods

#### 4.3.1. Structural and Spectroscopic Characterization of TOCs

The vibrational spectra of the synthesized compounds (crystals) were registered using: (a) IR spectrophotometry (Perkin Elmer Spectrum 2000 FTIR spectrophotometer (PerkinElmer Inc., Waltham, MA, USA) (400–4000 cm^−1^ range, KBr pellets)), and (b) Raman spectroscopy (RamanMicro 200 spectrometer (PerkinElmer Inc., Waltham, MA, USA)). The Raman spectra were recorded using a laser with the wavelength 785 nm, with a maximum power of 350 mW, in the range 200–3200 cm^−1^, with a 20 × 0.40/FN22 objective lens and an exposure time of 15 s each time. The ^13^C NMR spectra in the solid phase were recorded on a Bruker Advance 700 (Madison, WI, USA) 700 MHz spectrometer, with a spectral width of 76,923.08 Hz and 4096 complex points. Elemental analyses were performed on an Elemental AnalyserVario Macro CHN (ElementarAnalysensysteme GmbH, Langenselbold, Germany). 

#### 4.3.2. Single Crystal X-ray Diffraction Measurement 

The diffraction data were collected at 100 K on a Rigaku XtaLAB Synergy (Dualflex) diffractometer (Rigaku Inc., Wilmington, MA, USA) with a HyPix detector with a monochromated CuKα X-ray source (λ = 1.54184 Å). The data processing and the numerical absorption correction were performed using CrysAlis Pro [77]. The structure was using direct methods and refined by employing the full-matrix least-squares procedure on F^2^ (SHELX-97 [78]). Heavy atoms were refined with anisotropic displacement parameters, whereas hydrogen atoms were assigned at calculated positions, with thermal displacement parameters fixed to a value of 20% or 50% higher than those of the corresponding carbon atoms. A disorder was observed for the O31 (0.5:0.5) and O111 (0.6:0.4) isopropionate anions. Some restraints on geometry (DFIX) and thermal parameters (ISOR) of those disordered anions were applied to assure a stable refinement process. All figures were prepared in DIAMOND [79]. The results of the data collections and refinement have been summarized in Table 4; selected bond lengths and angles are presented in Table S1. CCDC 2310326 contains the supplementary crystallographic data for (**1**). These data can be obtained free of charge from The Cambridge Crystallographic Data Center via www.ccdc.cam.ac.uk.data_request/cif (accessed on 25 November 2023).

#### 4.3.3. X-ray Diffraction of Powders

Powder experiments were performed using a Rigaku XtaLAB Synergy (Dualflex) diffractometer with a HyPix detector and a monochromated CuKα X-ray source (λ = 1.54184 Å), working in the powder diffraction mode. The data were collected in the range 4–50° 2θ, with an exposure time 240 s per frame.

#### 4.3.4. Characterization of PMMA + TOCs Composite Materials

The chemical composition of the produced composite films was determined using an energy-dispersive X-ray spectrometer (EDS, Quantax 200 XFlash 4010, Bruker AXS, Karlsruhe, Germany). The maximum of absorption for the PMMA + (**1**) samples was also registered using the UV–Vis DRS method. The resulting composite materials had a thickness of approximately 50 µm and were characterized using Raman. 

#### 4.3.5. The Electron Paramagnetic Resonance (EPR) Spectroscopy

Formation of reactive oxygen species on the surface of the obtained samples was confirmed using EPR. The spectra were recorded using an X band EPR SE/X-2541M spectrometer (Radiopan, Poznań, Poland) with a 100 kHz modulation. The microwave frequency was monitored with a frequency meter. The magnetic field was measured with an automatic NMR-type JTM-147 magnetometer (Radiopan, Poznań, Poland). Measurement conditions: microwave frequency: ca. 9.33 GHz; modulation amplitude: 0.25–1 mT; sweep: 20–50 mT; sweep time: 4 min.; time constant: 0.1 s; receiver gain: 4 × 10^5^. The measurements were performed for the powder of the substrate and the cut films of PMMA + TOCs composites, at room temperature.

### 4.4. Studies of the Biological Activity of Synthesized Materials

#### 4.4.1. Antimicrobial Activity of PMMA + (**1**) Composites and Powder (**1**)

The antimicrobial activity of the samples was determined against Gram-negative (*Escherichia coli* ATCC 25922, *Escherichia coli* ATCC 8739) and Gram-positive (*Staphylococcus aureus* ATCC 25923, *Staphylococcus aureus* ATCC 6538) bacteria and *Candida albicans* ATCC 10231. Prior to antimicrobial study, the tested PMMA + TOCs foils (20 × 20 mm) were sterilized using UVC for 15 min on both sides, subsequently treated with visible indoor light, placed in the 12-well plates with 1 mL of microbial inoculum (1.0–4.7 × 10^6^ c.f.u. mL^−1^) in sterile deionized water, and incubated for 24 h at 37 °C in a humid atmosphere under gently shaking (80 r.p.m.) conditions. 

Microbial inoculum with a density of 0.5 McFarland (approximately 1.5 × 10^8^ c.f.u. mL^−1^) were prepared in sterile distilled water from cultures of bacterial strains and *C. albicans* grown in tryptic soy broth (TSB, Becton Dickinson, Heidelberg, Germany) and Sabouraud dextrose broth (SDB, Becton Dickinson), respectively, for 24 h at 37 °C, under shaking conditions (120 rpm). Each of the microbial inoculum were diluted 100 times prior to use. 

The control was the suspension of test microorganisms in the well, without test samples. After incubation, inoculum was collected from the wells, diluted ten-fold, and spread (100 µL) on appropriate medium in Petri dishes. The plates were incubated for 24 h at 37 °C, and colony forming units (c.f.u) were counted on the inoculated plates. The concentration of microorganisms was calculated per one mL.

The antimicrobial activity of the samples of (**1**) were determined by suspending them in microbial inoculum in sterile deionized water to obtain suspensions with concentrations of 2, 5, 10, and 20% (*w*/*v*) and gently (20 r.p.m) mixing them using the rotary shaker (Biosan, Riga, Latvia) for 24 h at 37 °C. 

The microbial inoculum after treatment with (**1**) samples was diluted ten-fold in sterile deionized water, and each dilution (1000 µL) was aseptically mixed with 20 mL of appropriate medium in Petri plates and incubated at 37 °C for 24 h. The colony forming units (c.f.u) were counted and the final concentration of the microorganisms was calculated per one mL.

The antimicrobial activity of the powder and the composites was determined based on the reduction (R) index, calculated according to the formula: R = Ut − At, where Ut is the common logarithm of the number of microorganisms in the inoculum, and At is the common logarithm of the number of microorganisms in the treated inoculum. R ≥ 2 and R ≥ 4 determine the biocidal activity of (**1**) in the studied form of the composite (antimicrobial activity of the surface) and suspension samples, respectively.

#### 4.4.2. Assessment of Material Cytotoxicity

The L929 murine fibroblast cell line (NCTC clone 929) was purchased from American Type Culture Collection (Manassas, VA, USA). The cells were cultured in Dulbecco′s Modified Eagle′s Medium (DMEM) supplemented with 10% heat-inactivated fetus bovine serum and antibiotics (100 µg/mL of streptomycin and 100 IU/mL of penicillin) at 37 °C in an atmosphere of 5% CO_2_. All reagents used for cell culture were purchased from VWR International (Radnor, PA, USA).

The specimens were cut into squares measuring 6 mm × 6 mm and were sterilized using UV irradiation for 30 min for each side of the samples before testing.

For evaluation of potential cytotoxicity of the tested materials, the samples were placed in individual wells of 24-well plates, followed by seeding of the cell suspension (1 × 10^4^ cells suspended in a 25 µL of culture medium). Then, the cells were incubated for 4 h at 37 °C and 5% CO_2_ to allow the cells to attach to the surface of the materials before flooding with 1 mL of DMEM medium. The L929 cells were incubated on the specimens for 24 and 72 h at 37 °C and 5% CO_2_.

After incubation, the samples were transferred to new wells of 24-well plates, and 500 µL of the MTT (tetrazolium salt 3-[4,5-dimethylthiazol-2-yl]-2,5-diphenyltetrazolium bromide purchased from Merck KGaA (Darmstadt, Germany)) solution, prepared in culture medium without phenol red at a final concentration 0.5 mg/mL, was added to each well and kept in an incubator for 3 h. Then, the MTT solution was aspirated, and 500 µL of dimethyl sulfoxide was added to each well, followed by measurement of the absorbance at 570 nm with the subtraction of the 630 nm background, using a Synergy HT microplate reader (BioTek Instruments, Winooski, VT, USA). The MTT assays were repeated in four separate experiments. All values are reported as means ± standard error (SEM). Statistical analysis of the data was performed using two-way analysis of variance (ANOVA) and a Duncan test to determine differences in cytotoxicity, with the level of significance set at *p* < 0.05.

The analysis of cell numbers growing on the sample surfaces, as well as cell morphology, was conducted using scanning electron microscopy (SEM; Quanta 3D FEG; Carl Zeiss, Göttingen, Germany). After 24 or 72 h, the cells were washed in PBS and fixed in 2.5% *w*/*v* glutaraldehyde for 4 h at 4 °C. Subsequently, the specimens were dehydrated in ethanol at increasing concentrations (50%, 75%, 90%, and 100%) for 10 min for each concentration at room temperature. Finally, the specimens were dried and stored at room temperature until the SEM analysis was performed.

## 5. Conclusions

Crystals of an oxo complex with the general formula [Ti_8_O_2_(O^i^Pr)_20_(man)_4_] (**1**) were successfully isolated from the reaction mixture of titanium isopropoxide and mandelic acid, mixed in a molar ratio of 4:1 at room temperature, using tetrahydrofuran as a solvent. Structural analysis revealed that the {Ti_8_O_2_} core comprises a central {Ti_4_O_2_} unit and two side dimers ({Ti_2_}), stabilized by two μ_3_-O bridges, bridge-forming anions, and a five-membered chelate ring.

Spectral tests, including XRD, IR, and Raman spectroscopy, confirmed the structural stability of compound (**1**) in both aqueous environments and after incorporation into a polymer matrix. EPR spectroscopy facilitated the detection of reactive oxygen species (ROS) on the surface of the grains (**1**) (in O_2_^−^ form), as well as in the PMMA + (**1**) composite film (in O^−^ and O_2_^−^ forms).

Biocidal assessments of compound (**1**) and the PMMA + (**1**) composite were conducted against *E. coli* and *S. aureus* bacteria strains, as well as *Candida albicans fungi*. The antibacterial effect was observed for a suspension containing as little as 2% of (**1**). A suspension containing 20% of (**1**) exhibited biocidal activity against both bacteria and fungi. The bactericidal activity of the composite PMMA + (**1**) systems was slightly weaker; moreover, they did not show a biocidal effect against fungi.

The observed significant antimicrobial activity of oxo complex (**1**) could stem from the synergetic effects associated with ROS generation and the existence of a chelating ring within its structure. Incorporating (**1**) into the polymer matrix restricted its direct interaction with microorganisms, consequently diminishing its antimicrobial efficacy. However, in such instances, the mechanisms linked to ROS generation remain operational. All PMMA + (**1**) materials demonstrated non-cytotoxic characteristic towards L929 fibroblasts. 

In summary, the test results from our investigations affirm that compound (**1**) fulfills its intended objectives, namely, possessing antibacterial properties, while being non-cytotoxic. This compound can be utilized as an antibacterial coating in public facilities or hospitals to mitigate the risk of bacterial infections.

## Figures and Tables

**Table 1:** SEM EDX data for composites PMMA + (**1**). All values are given in mass percent (%).

Composite	C	O	Al	Ti
PMMA	26.10	72.23	1.67	-
PMMA + (**1**) 2 wt.%	28.27	71.00	0.45	0.27
PMMA + (**1**) 5 wt.%	24.75	66.45	0.59	8.21
PMMA + (**1**) 10 wt.%	19.02	67.24	0.52	13.22
PMMA + (**1**) 20 wt.%	18.77	58.68	0.48	22.07

**Table 2:** EPR data for powdered TOC and PMMA + (**1**) composite samples. The samples were exposed to visible light prior to measurement.

Sample	g-Factor	Species
(**1**)	2.025, 2.011, 2.003	O_2_^−^
	1.992	Ti(III)
PMMA	-	-
PMMA + (**1**) 5 wt.%	2.010, 2.002	O_2_^−^
	2.005, 2.000	O^−^
	1.992	Ti(III)
PMMA + (**1**) 10 wt.%	2.025, 2.010, 2.000	O_2_^−^
	2.016	O^−^
	1.992	Ti(III)
PMMA + (**1**) 20 wt.%	2.025, 2.010, 2.002	O_2_^−^
	2.016, 2.005, 2.000	O^−^
	1.992, 1.972	Ti(III)

**Table 3:** Antimicrobial activity of TOC and PMMA + n(**1**) composites; value R ≥ 2 determines biocidal activity for the composites and R ≥ 3 for the (**1**) suspension.

No.		Microorganisms				
	Samples	*E. coli* ATCC 8739	*E. coli* ATCC 25922	*S. aureus* ATCC 6538	*S. aureus* ATCC 25923	*C. albicans* ATCC 10231
1	(**1**) 2 wt.%	6.0 (>99.99%)	6.0 (>99.99%)	4.2 (>99.99%)	5.4 (>99.99%)	0 (0%)
2	(**1**) 5 wt.%	6.0 (>99.99%)	5.7 (>99.99%)	5.9 (>99.99%)	6.0 (>99.99%)	0.3 (53.20%)
3	(**1**) 10 wt.%	6.0 (>99.99%)	6.0 (>99.99%)	6.2 (>99.99%)	6.0 (>99.99%)	0.9 (87.45%)
4	(**1**) 20 wt.%	6.0 (>99.99%)	6.0 (>99.99%)	6.5 (>99.99%)	5.7 (>99.99%)	6.7 (>99.99%)
5	PMMA	none (0%)	none (0%)	none (0%)	none (0%)	none (0%)
6	PMMA + (**1**) 2 wt.%	2.0 (99.00%)	3.1 (>99.90%)	4.7 (>99.99%)	5.1 (>99.99%)	+0.9 (+87.41%)
7	PMMA + (**1**) 5 wt.%	3.1 (>99.90%)	4.9 (>99.99%)	4.7 (>99.99%)	5.1 (>99.99%)	+0.82 (+84.86%)
8	PMMA + (**1**) 10 wt.%	4.9 (>99.99%)	3.9 (>99.90%)	4.7 (>99.99%)	5.1 (>99.99%)	+0.8 (+84.20%)
9	PMMA + (**1**) 20 wt.%	4.9 (>99.99%)	4.7 (>99.99%)	4.7 (>99.99%)	5.1 (>99.99%)	+0.7 (+80.05%)

**Table 4:** Crystal data and structure refinement for (**1**).

Empirical formula	C_92_H_164_O_34_Ti_8_ (**1**)
Formula weight	2197.42
Temperature	100(2) K
Wavelength [Å]	1.54184
Crystal system	Monoclinic
Space group	P2_1_/c
Unit cell dimensions [Å] and [°]	a = 19.9172(6)
	b = 12.3935(3)
	c = 24.4651(8)
	α = 90
	β = 111.483(4)
	γ = 90
Volume [Å^3^]	5619.5(3)
Z, calculated density [Mg/m^3^]	2, 1.299
Absorption coefficient [mm^−1^]	5.193
F(000)	2328
Crystalsize [mm^3^]	0.220 × 0.180 × 0.080
Theta range for data collection [°]	2.384 to 74.492
Index ranges	−24 ≤ h *≤* 24
	−15 *≤* k *≤* 14
	−30 *≤* l *≤* 21
Reflections collected/unique	44,138/11,218 [R(int) = 0.0943
Completeness to theta	67.684° 99.9%
Absorption correction	Gaussian
Max. and min. transmission	1.000 and 0.414
Refinement method	Full-matrix least-squares on F2
Data/restraints/parameters	11,218/35/654
Goodness-of-fit on F2	1.054
Final R indices [I > 2sigma(I)]	R1 ^a^ = 0.0865, wR2 ^b^ = 0.2451
R indices (all data)	R1 ^a^ = 0.1092, wR2 ^b^ = 0.2674
Largest diff. peak and hole	0.766 and −0.915 e·Å^−3^

^a^ R1 = ∑‖F_0_| − |F_c_|/∑|F_0_|, ^b^ wR2 = [∑w(F_0_^2^ − F_c_^2^)^2^/∑(w(F_0_^2^)^2^)]^1/2^.

## Data Availability

Crystallographic data have been deposited at The Cambridge Crystallographic Data Center via www.ccdc.cam.ac.uk.data_request/cif accessed on 25 November 2023. Other data are unavailable due to their potential application meaning.

## Supplementary Materials

The following supporting information can be downloaded at: https://www.mdpi.com/article/10.3390/molecules29081736/s1, Figure S1: The topological analysis of the cluster with a {Ti_4_O_2_} core (left) [7] (a), and {Ti_8_O_2_} (**1**) (right) (b), performed in TOPS; Figure S2: A 3D-deformation density map for (**1**) showing the presence of charge depletion regions (in red) and charge concentration regions (in blue), mapped using Crystal Explorer 21.5. The isosurfaces are drawn at 0.008 eau−3. The green circles show two of four chelate rings present in this molecule. In all of them, the same feature occurs—the blue color prevails, pointing at charge concentration in this region. The wavefunction was calculated at the level B3LYP/6-31G(d,p) References [80,81,82,83]; Figure S3: EPR spectra obtained for the powdered sample of (**1**) (a) and a cut foil of PMMA + (**1**) 20 wt.% (b). The experimental conditions were as follows: room temperature, microwave frequencies of 9.31648 (a) and 9.32357 (b) GHz; modulation amplitude of 1 mT; sweep width of 20 mT; sweep time of 4 min; time constant of 0.1 s; receiver gain of 4 × 10^5^; Table S1: Selected bond lengths [Å] and bond angles [°] in (**1**); Table S2. Coordination modes in (**1**).
