# Supplementary material for: Structural Characterization and Bioactivity of a Titanium(IV)-Oxo Complex Stabilized by Mandelate Ligands
Source: Molecules. 2024 Apr 11;29(8):1736. doi: 10.3390/molecules29081736 (PMC11052117; doi:10.3390/molecules29081736)
Supplement: Supplementary file 1 [file molecules-29-01736-s001.zip › molecules-2946565-supplementary.pdf]

Supplementary Information

Structural Characterization and Bioactivity of a Titanium(IV)-Oxo Complex Stabilized by Mandelate Ligands

Barbara Kubiak 1,*, Tadeusz Muzioł 1, Grzegorz Wrzeszcz 1, Aleksandra Radtke 1, Patrycja Golińska 2, Tomasz Jędrzejewski 3, Sylwia Wrotek 3 and Piotr Piszczek 1,*

1 Department of Inorganic and Coordination Chemistry, Faculty of Chemistry, Nicolaus Copernicus University in Toruń, Gagarina 7, 87-100 Toruń, Poland; tadeuszmuziol@wp.pl (T.M.); wrzeszcz@umk.pl (G.W.); aleksandra.radtke@umk.pl (A.R.)
2 Department of Microbiology, Faculty of Biological and Veterinary Sciences, Nicolaus Copernicus University in Toruń, Lwowska 1, 87-100 Toruń, Poland; golinska@umk.pl
3 Department of Immunology, Faculty of Biological and Veterinary Sciences, Nicolaus Copernicus University in Toruń, Lwowska 1, 87-100 Toruń, Poland; tomaszj@umk.pl (T.J.); wrotek@umk.pl (S.W.)
* Correspondence: basiak0809@gmail.com (B.K.); piszczek@umk.pl (P.P.)

Table S1. Selected bond lengths [Å] and bond angles [°] in (**1**).

	Distance [Å]		Angles [°]		Angles [°]
Ti1-O21	1.798(3)	O21-Ti1-O10	106.35(13)	O3-Ti2-O31^{i}	88.97(12)
Ti1-O10	1.867(3)	O21-Ti1-O31	95.00(13)	O10-Ti2-O31^{i}	75.77(11)
Ti1-O31	1.952(3)	O10-Ti1-O31	104.11(12)	O12-Ti2-O31^{i}	82.35(12)
Ti1-O1	2.055(3)	O21-Ti1-O1	93.57(13)	O81-Ti3-O71	93.59(19)
Ti1-O3	2.058(3)	O10-Ti1-O1	147.75(13)	O81-Ti3-O61	108.5(2)
Ti1-O10^{i}	2.074(3)	O31-Ti1-O1	98.98(12)	O71-Ti3-O61	99.92(18)
Ti1-Ti1^{i}	3.0086(13)	O21-Ti1-O3	92.88(13)	O81-Ti3-O13	171.30(16)
Ti1-Ti2	3.1036(10)	O10-Ti1-O3	78.25(11)	O71-Ti3-O13	94.42(16)
Ti1-Ti2^{i}	3.1940(10)	O31-Ti1-O3	170.73(13)	O61-Ti3-O13	73.42(15)
Ti2-O41	1.769(3)	O1-Ti1-O3	75.62(11)	O81-Ti3-O11	100.03(18)
Ti2-O51	1.807(3)	O21-Ti1-O10^{i}	171.33(13)	O71-Ti3-O11	93.70(16)
Ti2-O3	2.006(3)	O10-Ti1-O10^{i}	80.61(12)	O61-Ti3-O11	147.34(15)
Ti2-O10	2.040(3)	O31-Ti1-O10^{i}	78.06(12)	O13-Ti3-O11	76.06(13)
Ti2-O12	2.054(3)	O1-Ti1-O10^{i}	82.53(11)	O81-Ti3-O2^{i}	82.89(16)
Ti2-O31^{i}	2.091(3)	O3-Ti1-O10^{i}	93.64(11)	O71-Ti3-O2^{i}	173.50(17)
Ti3-O81	1.784(4)	O41-Ti2-O51	97.93(16)	O61-Ti3-O2^{i}	86.41(14)
Ti3-O71	1.795(4)	O41-Ti2-O3	95.43(15)	O13-Ti3-O2^{i}	88.81(13)
Ti3-O61	1.933(4)	O51-Ti2-O3	95.79(13)	O11-Ti3-O2^{i}	81.59(12)
Ti3-O13	2.038(4)	O41-Ti2-O10	98.47(14)	O101-Ti4-O111	101.9(3)
Ti3-O11	2.070(3)	O51-Ti2-O10	162.13(14)	O101-Ti4-O91	111.3(3)
Ti3-O2^{i}	2.172(3)	O3-Ti2-O10	75.65(11)	O111-Ti4-O91	100.0(3)
Ti3-Ti4	3.2486(15)	O41-Ti2-O12	91.80(15)	O101-Ti4-O13	118.1(3)
Ti4-O101	1.773(5)	O51-Ti2-O12	96.26(14)	O111-Ti4-O13	91.02(19)
Ti4-O111	1.787(5)	O3-Ti2-O12	164.96(12)	O91-Ti4-O13	125.55(19)
Ti4-O91	1.801(5)	O10-Ti2-O12	90.27(12)	O101-Ti4-O61	90.6(3)
Ti4-O13	1.979(3)	O41-Ti2-O31^{i}	171.70(14)	O111-Ti4-O61	161.70(19)
Ti4-O61	2.098(4)	O51-Ti2-O31^{i}	88.60(14)	O91-Ti4-O61	87.5(2)
				O13-Ti4-O61	71.20(14)

Table S2. Coordination modes in (**1**).

	Coordination sphere content – number of atoms involved in Ti(IV) binding					
Central atom	Coordination number	mandelate		$iPrO^-$ anion		Oxo anion
		$RCOO^-$	RO^-	terminal	bridging	
Ti1	6	1	**1**	1	1	2
Ti2	6	1	1	2	1	1
Ti3	6	2	**1**	2	1	-
Ti4	5	-	1	3	1	-

In bold are marked hydroxyl groups involved in five-membered chelate ring with one oxygen atom from carboxylic group

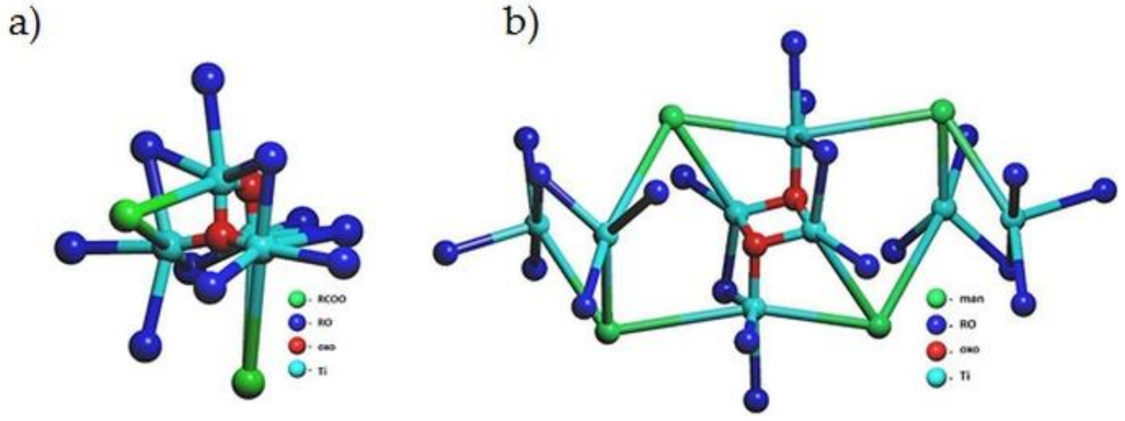

Figure S1. The topological analysis of the cluster with $\{Ti_4O_2\}$ core ([7] left) (a), and $\{Ti_8O_2\}$ (**1**) (this paper, right) cores performed in TOPOS with titanium cations in cyan, oxo anions in red, mandelate (carboxylate) anions in green and propionate anions in blue.

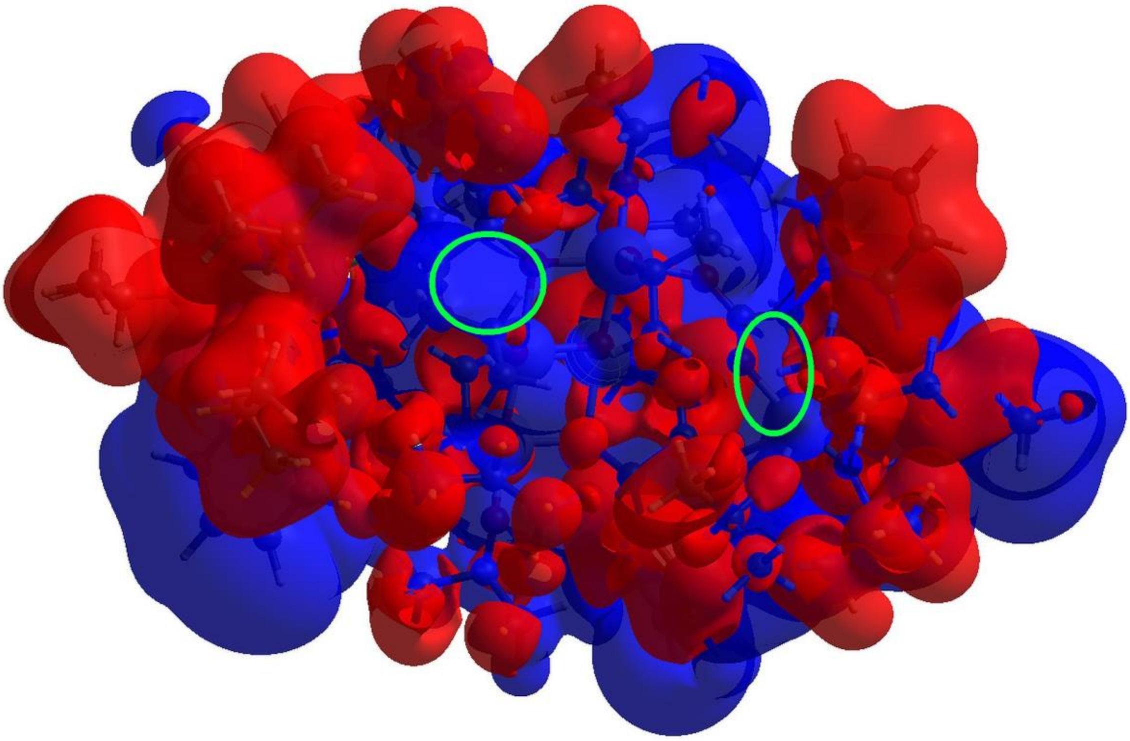

Figure S2. 3D-deformation density map for (**1**) showing the presence of charge depletion regions (in red) and charge concentration regions (in blue), mapped using Crystal Explorer 21.5. The isosurfaces are drawn at 0.008 eau−3. The green circles show two of four chelate rings present in this molecule. In all of them the same feature occurs – the blue color prevails pointing at charge concentration in this region. The wavefunction was calculated at the level B3LYP/6-31G(d,p) [80-83].

Reference:

[80] Becke, A.D. Density-functional thermochemistry. III. The role of exact exchange. J. Chem. Phys. 1993, 98, 5648–5652.
[81] Stephens, P.J.; Devlin, F.J.; Chabalowski, C.F.; Frisch, M.J. Ab Initio Calculation of Vibrational Absorption and Circular Dichroism Spectra Using Density Functional Force Fields. J. Phys. Chem. 1994, 98, 11623–11627.
[82] Becke, A.D. Density-functional exchange-energy approximation with correct asymptotic behavior. Phys. Rev. A 1988, 38, 3098–3100.
[83] Lee, C.; Yang, W.; Parr, R.G. Development of the Colle-Salvetti correlation-energy formula into a functional of the electron density. Phys. Rev. B 1988, 37, 785–789.

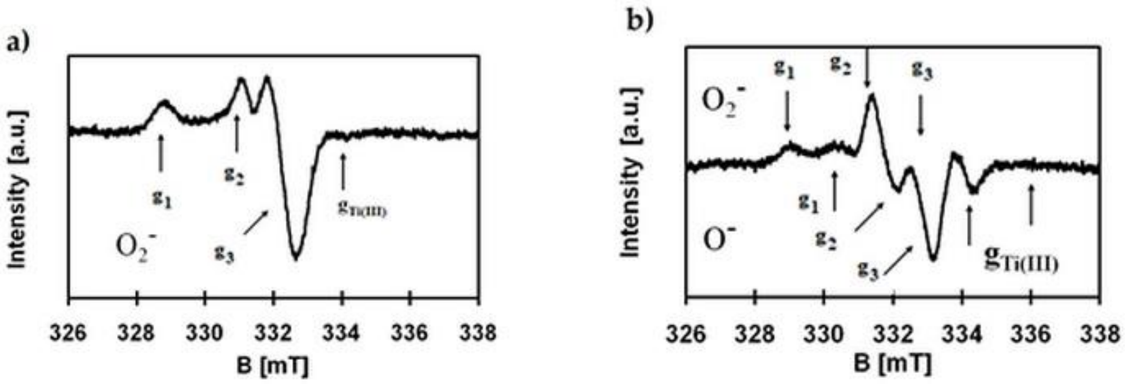

Figure S3. EPR spectra of powdered TOCs (a) and PMMA + (**1**) wt.20% composite (b) of TOCs. Some experimental conditions: room temperature, microwave frequency: 9.31648 GHz (a) 9.32357 GHz (b); modulation amplitude: 1 mT; sweep: 20 mT; sweep time: 4 min.; time constant: 0.1 s; receiver gain: 4 x10^{5} .
